# Improved YOLOv4 recognition algorithm for pitaya based on coordinate attention and combinational convolution

**DOI:** 10.3389/fpls.2022.1030021

**Published:** 2022-10-18

**Authors:** Fu Zhang, Weihua Cao, Shunqing Wang, Xiahua Cui, Ning Yang, Xinyue Wang, Xiaodong Zhang, Sanling Fu

**Affiliations:** ^1^ College of Agricultural Equipment Engineering, Henan University of Science and Technology, Luoyang, China; ^2^ Collaborative Innovation Center of Machinery Equipment Advanced Manufacturing of Henan Province, Henan University of Science and Technology, Luoyang, China; ^3^ School of Electrical and Information Engineering, Jiangsu University, Zhenjiang, China; ^4^ Key Laboratory of Modern Agricultural Equipment and Technology of Ministry of Education, Jiangsu University, Zhenjiang, China; ^5^ College of Physical Engineering, Henan University of Science and Technology, Luoyang, China

**Keywords:** improved YOLOv4, GhostNet, coordinate attention, improved combinational convolution module, target recognition

## Abstract

Accurate recognition method of pitaya in natural environment provides technical support for automatic picking. Aiming at the intricate spatial position relationship between pitaya fruits and branches, a pitaya recognition method based on improved YOLOv4 was proposed. GhostNet feature extraction network was used instead of CSPDarkNet53 as the backbone network of YOLOv4. A structure of generating a large number of feature maps through a small amount of calculation was used, and the redundant information in feature layer was obtained with lower computational cost, which can reduce the number of parameters and computation of the model. Coordinate attention was introduced to enhance the extraction of fine-grained feature of targets. An improved combinational convolution module was designed to save computing power and prevent the loss of effective features and improve the recognition accuracy. The Ghost Module was referenced in Yolo Head to improve computing speed and reduce delay. Precision, Recall, F1, AP, detection speed and weight size were selected as performance evaluation indexes of recognition model. 8800 images of pitaya fruit in different environments were used as the dataset, which were randomly divided into the training set, the validation set and the test set according to the ratio of 7:1:2. The research results show that the recognition accuracy of the improved YOLOv4 model for pitaya fruit is 99.23%. Recall, F1 and AP are 95.10%, 98% and 98.94%, respectively. The detection speed is 37.2 frames·s^-1^, and the weight size is 59.4MB. The improved YOLOv4 recognition algorithm can meet the requirements for the accuracy and the speed of pitaya fruit recognition in natural environment, which will ensure the rapid and accurate operation of the picking robot.

## 1 Introduction

Pitaya belongs to the cactus family, which has many branches and extends. The edges of the leaves are winged, wavy or crenellated, which make the harvesting process time-consuming and labor-intensive. Rapid and accurate recognition of pitaya fruit is a prerequisite for automatic picking by agricultural robots. Therefore, it has important research significance and application value to improve operation efficiency (Tang et al., 2020; Huang et al., 2021; Ye et al., 2021).

At present, scholars have carried out researches on the recognition of target fruits and vegetables based on traditional image processing technology (Zeng et al., 2021; Miao et al., 2021). Han et al. (2021) established an automatic quantification system based on HSV space model for the segmentation of amygdalus mira seeds, and the accuracy rate was 99.7%. Zhang et al. (2019) converted RGB image into a Lab space model, and used Hough circle transform to count the number of fruits, and the recognition accuracy was 94.01%. Zhang et al. (2020) proposed a pomegranate fruit recognition and classification method based on support vector machine and multi-feature fusion, and its classification accuracy was 75%. Liu et al. (2019) extracted the color and shape features of ripe apples to realize apple recognition, and Recall was 89.8%. Chu et al. (2019) proposed a method for identifying spherical fruits based on machine vision, and the recognition accuracy was over 95%. The above methods achieve fruits recognition by extracting the color, shape and texture features. However, those methods have problems such as long detection time, poor robustness, and low recognition accuracy, which are difficult to meet the recognition accuracy of target fruits in intricate environments (Liu et al., 2018; Tan et al., 2018; Xue et al., 2018; Zhao et al., 2020).

In recent years, the convolutional neural network (CNN) has been widely used in target recognition and detection (Lv et al., 2019; Cao et al., 2021; Zhang K. et al., 2021), which is mainly divided into two categories. One is the two-stage target detection method represented by region-CNN (RCNN) (Girshick et al., 2014), Fast RCNN (Girshick, 2015), Faster RCNN (Ren et al., 2015), etc., the steps for these methods are to obtain the target proposal box firstly, and then classified it in the proposal box. Zhu et al. (2020) proposed an improved Faster RCNN algorithm based on the botanical characteristics of lycium barbarum flowering period and fruit ripening period, and its average accuracy reached 74%. Yan et al. (2019) proposed an improved Faster RCNN algorithm to identify prickly pears, and its average recognition accuracy reached 92.01%. Zhang W. et al. (2021) proposed an improved Faster RCNN algorithm to identify tomatoes, and its average recognition accuracy reached 95.2%. Such algorithms have a long training time and slow detection speed. Another is the one-stage target detection method represented by SSD (Liu et al., 2016), YOLO (Redmon et al., 2016), etc., which completes the target proposal box and classification label in the same network. Yi et al. (2021) proposed a YOLOv4 model based on feature recursive fusion to identify citrus, and its detection accuracy reached 94.6%. Wang et al. (2021) proposed I-YOLOv4-Tiny target detection network, and introduced convolutional attention module to identify blueberry fruit in different environments, and its average accuracy reached 97.30%. Zhang F. et al. (2021) proposed an improved YOLOv4-LITE target detection algorithm to identify cherry tomatoes, and its average accuracy reached 99.15%. Li et al. (2021) used MobileNetV2 as YOLOv3 backbone network and introduced M-Res2Net module to identify grape fruit, and its average accuracy reached 81.2%. Xiong et al. (2020) proposed Des-YOLOv3 target detection network to identify ripe citrus at night, and its average accuracy reached 90.75%. Wu et al. proposed an improved YOLOv3 model based on clustering optimization and a new YOLOv5-B model to obtain target information and improve the accuracy and speed of small target detection (Wu et al., 2021; Wu et al., 2022). Tang et al. (2022) proposed a model method YOLO-Oleifera for Camellia oleifera fruit detection based on the YOLOv4-tiny model, which realize the learning of the characteristic information of Camellia oleifera fruit. Li et al. (2022) built a new type of agricultural machinery intelligent design system integrating image processing and knowledge reasoning, which provided a reference for intelligent design to guide actual production. In all, the above researches have problems such as complex calculation, large consumption, which are difficult to meet the rapid and accurate operation of picking robot on the target fruits in an intricate environment. Therefore, the recognition accuracy and speed need to be improved.

In order to solve the difficulty of identifying and picking pitaya fruit in natural environment, this paper proposed an improved YOLOv4 recognition algorithm that integrated coordinate attention and combinational convolution to improve the recognition speed and accuracy of pitaya fruit.

## 2 Test materials and methods

### 2.1 Test data acquisition

**Figure 1:**
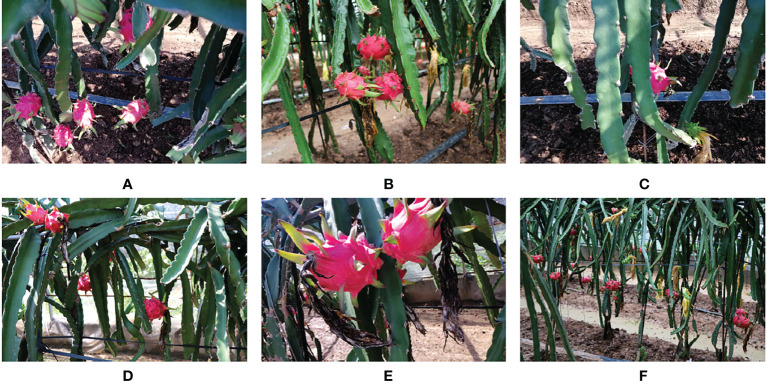
Some images of pitayas in greenhouse environment: **(A)** Mature pitayas in sunny days; **(B)** Mature pitayas in rainy days; **(C)** Occlusion of pitayas; **(D)** Adhesive pitayas; **(E)** Short distance pitayas; **(F)** Long distance pitayas.

The pitaya fruit images were collected in the greenhouse of Taiwan Fuhao farm, Mengjin district, Luoyang city, Henan province, which were collected under natural light conditions on rainy and sunny days. The image acquisition device is Canon (Canon EOS 750D) single-lens reflex camera, the image resolution is 6000 × 4000 pixels, and the format is JPG. In order to simulate the recognition system of picking robot, we chose to shoot from five angles: front, left, right, up and down. A total of 1,280 original images of pitaya fruit were collected, and 1,100 images of pitaya fruit growing environment in various natural environment including smooth light, backlight, overlap, occlusion, and adhesion were selected, as shown in **Figure 1**. In this research, the red heart “soft branch big red” pitaya fruit was used, which is suitable for planting in areas with a minimum temperature above 0 degrees in January all year round. They need to be tied and pruned at appropriate times. When the seedlings grow to the square (circle) position, they can be allowed to droop for early flowering and fruiting.

### 2.2 Data augmentation

Training a deep learning model requires a large number of data, and too small dataset will lead to the overfitting of neural network. Therefore, the data augmentation method was used to expand the number of samples. In this paper, methods such as translation transformation, random rotation, mirror flip, horizontal flip, brightness adjustment, salt and pepper noise were used for data enhancement. A total of 8800 images were obtained as the dataset.

### 2.3 Dataset preparation

We transfer the LabelImg image annotation software in Python to manually mark the rectangular box of the pitaya fruit in the image. The completely naked pitaya fruit is marked on the inside of its rectangular box, occluded or conglutinated pitaya fruit only needs to mark the exposed part of the image, and the pitaya fruit that appears less than 10% in the image is not marked. We set the target category to “pitaya”, and save it as.xml file after labeling all pitaya fruits in the image.

## 3 Pitaya fruit recognition network

### 3.1 YOLOv4 network model

**Figure 2:**
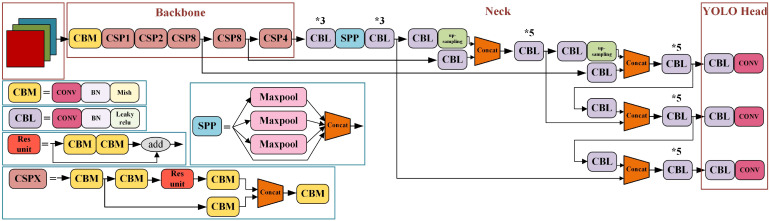
YOLOv4 network structure diagram. * means repeat the operation.

YOLO is a target recognition and localization algorithm based on deep neural network to achieve end-to-end prediction. YOLOv4 (Bochkovskiy et al., 2020) is an efficient and powerful target detection model combined with a large number of previous research techniques and combined innovation. YOLOv4 structure is shown in **Figure 2**.

The loss function of YOLOv4 consists of positive sample coordinate loss, positive sample confidence loss, negative sample confidence loss and positive sample classification loss. This paper uses Complete Intersection over Union (CIoU, taking into account the distance, overlap, scale and penalty terms between the target and the box, making the tar-get box regression more stable) as the loss function. CIoU makes the prediction box more consistent with the real box, so that the target box can be positioned accurately. It can also avoid the problem that the Intersection over Union (IoU, used to measure the degree of overlap between the prediction box and the real box in target detection) of the loss function is 0 which because the prediction box does not intersect the real box. The expression of CIoU is:


(1) $$CIoU=I\text{o}U-\frac{\rho^{2}(b,b^{gt})}{c^{2}}-\beta v$$

Among them, *ρ* ^2^(*b*,*b* ^*gt*^) represents the Euclidean distance between the prediction box and the center point of the real box, *c* represents the diagonal distance which tangential to the rectangular box outside the prediction box and the real box, *β* is a measure of aspect ratio consistency parameter, *v* is the trade-off parameter. The YOLOv4 loss function expression is:


(2) $$\begin{array}{c} L(object)=\lambda_{coord}\sum_{i=0}^{K\times K}\sum_{j=0}^{M}I_{ij}^{obj}(2-w_{i}\times h_{i})(1-CIOU)\sum_{i=0}^{K\times K}\sum_{j=0}^{M}I_{ij}^{obj}\\ -\sum_{i=0}^{K\times K}\sum_{j=0}^{M}I_{ij}^{obj}[\hat{C_{i}}\log(C_{i})+(1-\hat{C_{i}})\log(1-C_{i})]\\ -\lambda_{noobj}\sum_{i=0}^{K\times K}\sum_{j=0}^{M}I_{ij}^{noobj}[\hat{C_{i}}\log(C_{i})+(1-\hat{C_{i}})\log(1-C_{i})]\\ -\sum_{i=0}^{K\times K}\sum_{j=0}^{M}I_{ij}^{obj}\sum_{c\in classes}\left[\hat{P_{i}}(c)\log(P_{i}(c))+(1-\hat{P_{i}}(c))\log(1-P_{i}\left(c\right))\right] \end{array}$$

Among them, *λ*_*coord*_ is the weight coefficient of positive samples, $\sum_{i=0}^{K\times K}\sum_{j=0}^{M}$ represents traversing all prediction boxes, $I_{ij}^{obj}$ and $I_{ij}^{noobj}$ represent whether they are positive samples. 1 for positive samples, while 0 for others. *w*_*i*_ is the width of the center point of the prediction box, *h*_*i*_ is the height of the center point of the prediction box, $\hat{C_{i}}$ is the sample value, *C*_*i*_ is the predicted value, *λ*_*boobj*_ is the weight coefficient of negative samples.

### 3.2 Improved YOLOv4 network model

YOLOv4 uses CSPDarkNet53 backbone network. Although it has a good effect on object feature extraction, its own network parameters are too large, resulting in a slow recognition speed of YOLOv4. In addition, its model calculation is complex and requires a large amount of memory space. Accordingly, a fast, accurate and lightweight recognition model was proposed in this paper. Based on the traditional YOLOv4, GhostNet was used as the backbone network for feature extraction to reduce the computational complexity of the model, generate more feature maps and achieve rapid recognition of targets. To save computing power, learn more features and process more data in a shorter time, an improved combinational convolution module was used to replace the traditional combinational convolution at feature fusion, and the coordinate attention (CA, an attention mechanism that embeds location information into channel attention) was introduced. In order to compress the model, improve computing speed and reduce delay, Ghost Module was referenced in Yolo Head.

#### 3.2.1 GhostNet backbone network

**Figure 3:**
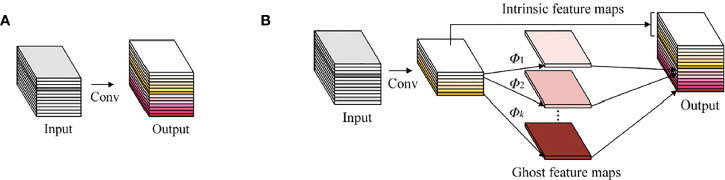
Comparison diagram between the convolutional layer and the Ghost Module: **(A)** The convolutional layer; **(B)** The Ghost Module.

GhostNet (Han et al., 2020) proposes a structure that generates a large number of feature maps only by a small amount of computation——Ghost Module. It generates feature maps through a series of linear operations. The feature maps generated by linear operations are called Ghost feature maps, and the operated feature maps are called intrinsic feature maps. The conventional convolution module is shown in **Figure 3A**, and the Ghost Mod-ule is shown in **Figure 3B**.

If the input feature map size is *h*_1_×*w*_1_×*c*, the output feature map size is *h*_2_×*w*_2_×*n*, the convolution kernel size is *k*×*k*, and the stride is *s*, then the conventional convolution and Ghost module FLOPs (measurable model complexity) are:


(3) $$FLOPs(a)=n\times h_{2}\times w_{2}\times c\times k\times k$$

(4) $$FLOPs(b)=\frac{n}{s}\times h_{2}\times w_{2}\times c\times k\times k\times (s-1)\times \frac{n}{s}\times h_{2}\times w_{2}\times k\times k$$

The ratio of the two is:


(5) $$\frac{FLOPs(a)}{FLOPs(b)}=\frac{n\times c}{\frac{n}{s}\times c\times (s-1)\times \frac{n}{s}}=\frac{s\times c}{c+s-1}\approx s$$

It can be seen that FLOPs of Ghost Module can be reduced to 1/*s* of the conventional convolution, which reduces the complexity of the model.

#### 3.2.2 Improved combinational convolution-CA module

**Figure 4:**
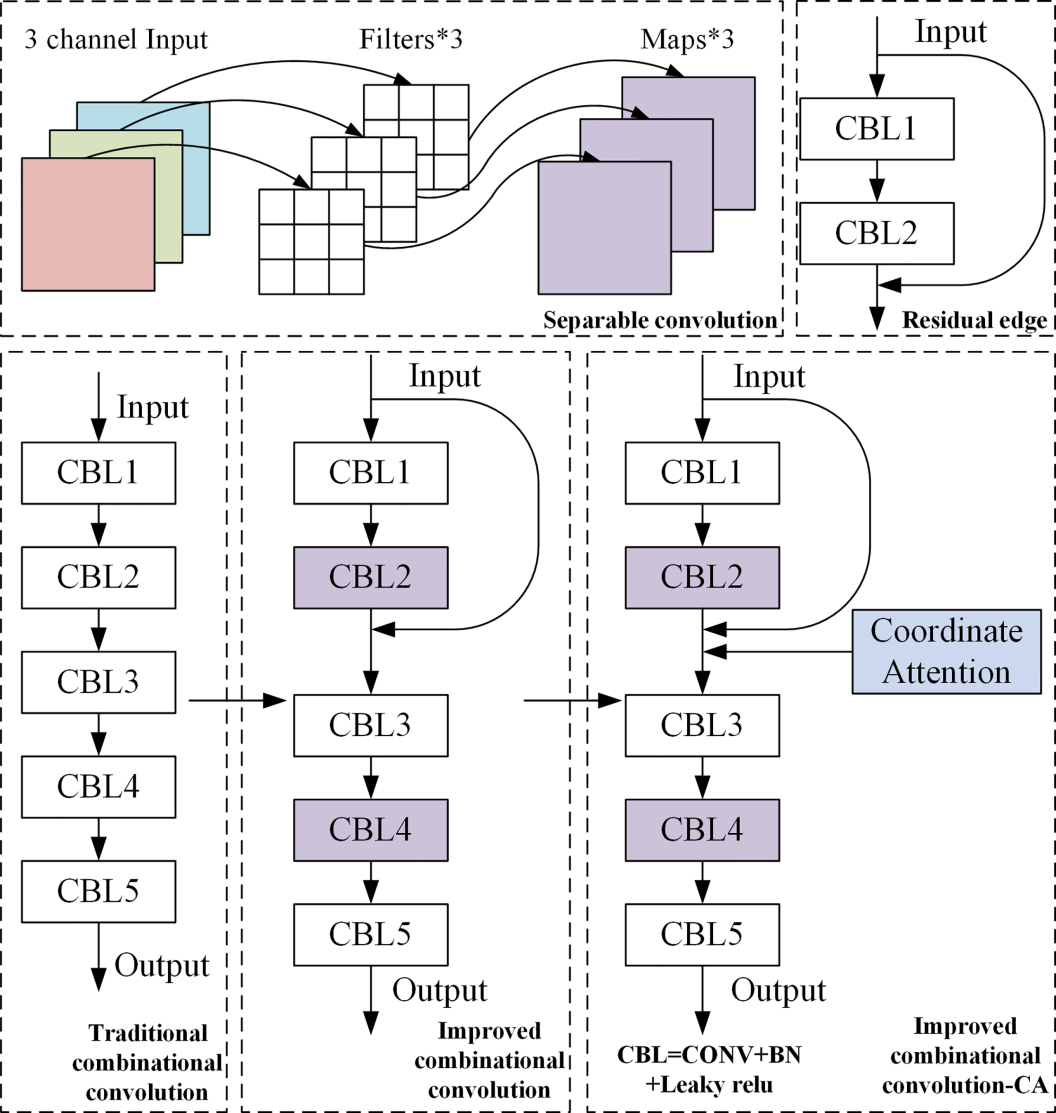
Structure diagram of improved combinational convolution-CA. * means repeat the operation.

Five combinational convolution improvements were made at feature fusion to generate an improved combinational convolution module. In other words, a separable convolution and a residual edge structure were introduced. Among them, the separable convolution can reduce the computational complexity of the network and run faster. The residual edge can improve the learning effect of the model, prevent the loss of effective features, and effectively solve the problem of gradient disappearance. Based on this, two conventional convolutions of the traditional combinational convolution were replaced by separable convolutions. Moreover, the residual edge was added next to the former two convolutions to obtain an improved combinational convolution module. To help the model locate and identify object of interest more accurately, CA was introduced. As a result, an improved combinational convolution-CA module was obtained, as shown in **Figure 4**.

**Figure 5:**
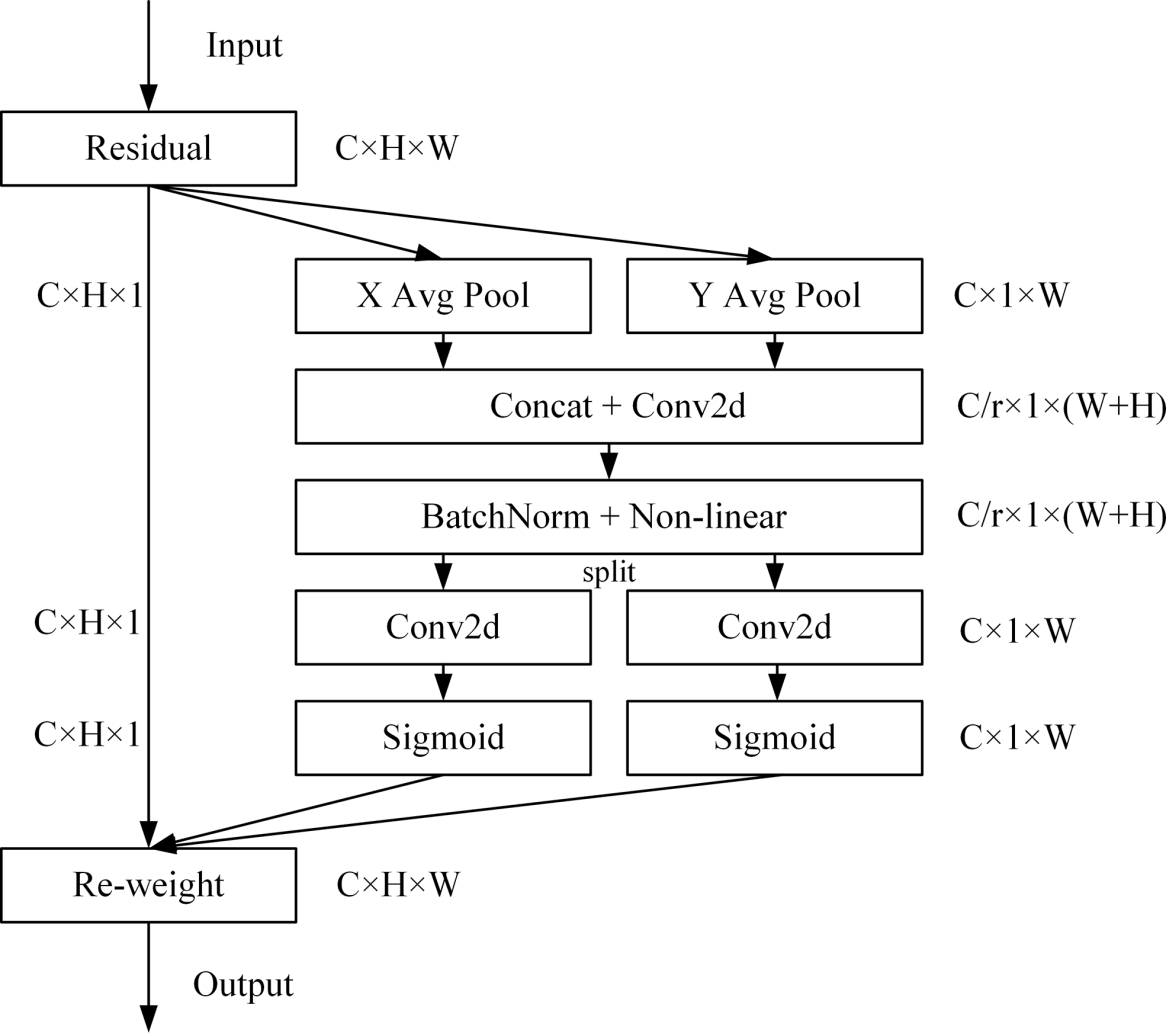
CA structure diagram.

CA (Hou et al., 2021) embeds location information into channel attention, which can reduce the attention to secondary information and enhance the extraction of fine-grained feature of targets to improve model accuracy and generalization performance. CA includes coordinate information embedding and coordinate attention generation. The structure is shown in **Figure 5**.

The coordinate information embedding operation corresponds to X Avg Pool and Y Avg Pool in the figure. For the input *X*, it uses the pooling kernel of dimension (*H*, 1) and (1, *W*) to encode each channel along the horizontal and vertical coordinate directions, and the output expression of the cth channel with height *h* is:


(6) $$z_{c}^{h}(h)=\frac{1}{W}\sum_{0\leq i<W}x_{c}(h,i)$$

Similarly, the output expression of the cth channel of width *w* is:


(7) $$z_{c}^{w}(w)=\frac{1}{H}\sum_{0\leq j<H}x_{c}(j,w)$$

For CA generation operation, the two feature maps generated by the previous module are concatenated firstly, and then use a shared 1×1 convolution transformation *F*_1_ and the expression is:


(8) $$f=\delta (F_{1}([z^{h},z^{w}]))$$

The generated *f*∈*R* ^*C*/*r*×(*H*+*W*)^ is the intermediate feature map of spatial information in horizontal and vertical directions, and *r* represents the down-sampling ratio to control the block size. Slice *f* into two separate tensors *f* ^*h*^∈*R* ^*C*/*r*×*H*^ and *f* ^*w*^∈*R* ^*C*/*r*×*W*^ along the spatial dimension, and then use two 1×1 convolutions *F*_*h*_ and *F*_*w*_ to transform the feature map *f* ^*h*^ and *f* ^*w*^ to the same number of channels as the input *X*, and get the following result:


(9) $$g^{h}=\sigma (F_{h}({\text{f}}^{h}))$$

(10) $$g^{w}=\sigma (F_{w}({\text{f}}^{w}))$$

Expanding *g* ^*h*^ and *g* ^*w*^ as the weight of attention. CA expression is:


(11) $$y_{c}(i,j)=x_{c}(i,j)\times g_{c}^{h}(i)\times g_{c}^{w}(j)$$

The calculation can capture the precise position relationship, and then locate the exact position of the object of interest more accurately, so as to help the model identify better.

#### 3.2.3 Improved combinational convolution-CA module at feature fusion

**Figure 6:**
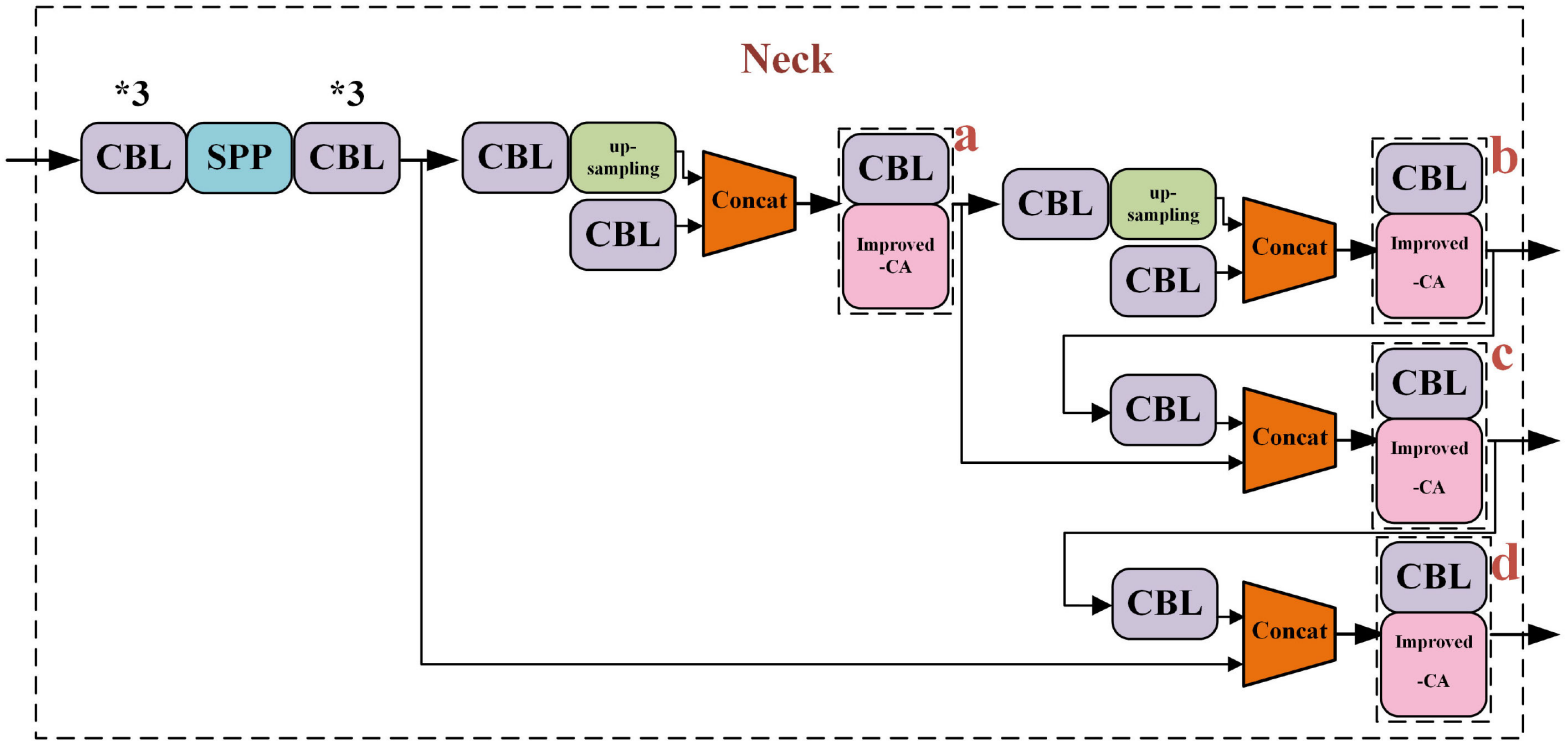
Improved combinational convolution-CA module at fusion.

The improved combinational convolution-CA module was applied to the feature fusion (a, b, c, d) section, and the improved combinational convolution-CA module was used at ac, bd, abcd respectively, replacing the original CBL module with the improved combinational convolution-CA module, as shown in **Figure 6**.

### 3.3 Model training and testing

#### 3.3.1 Test platform

**Figure 7:**
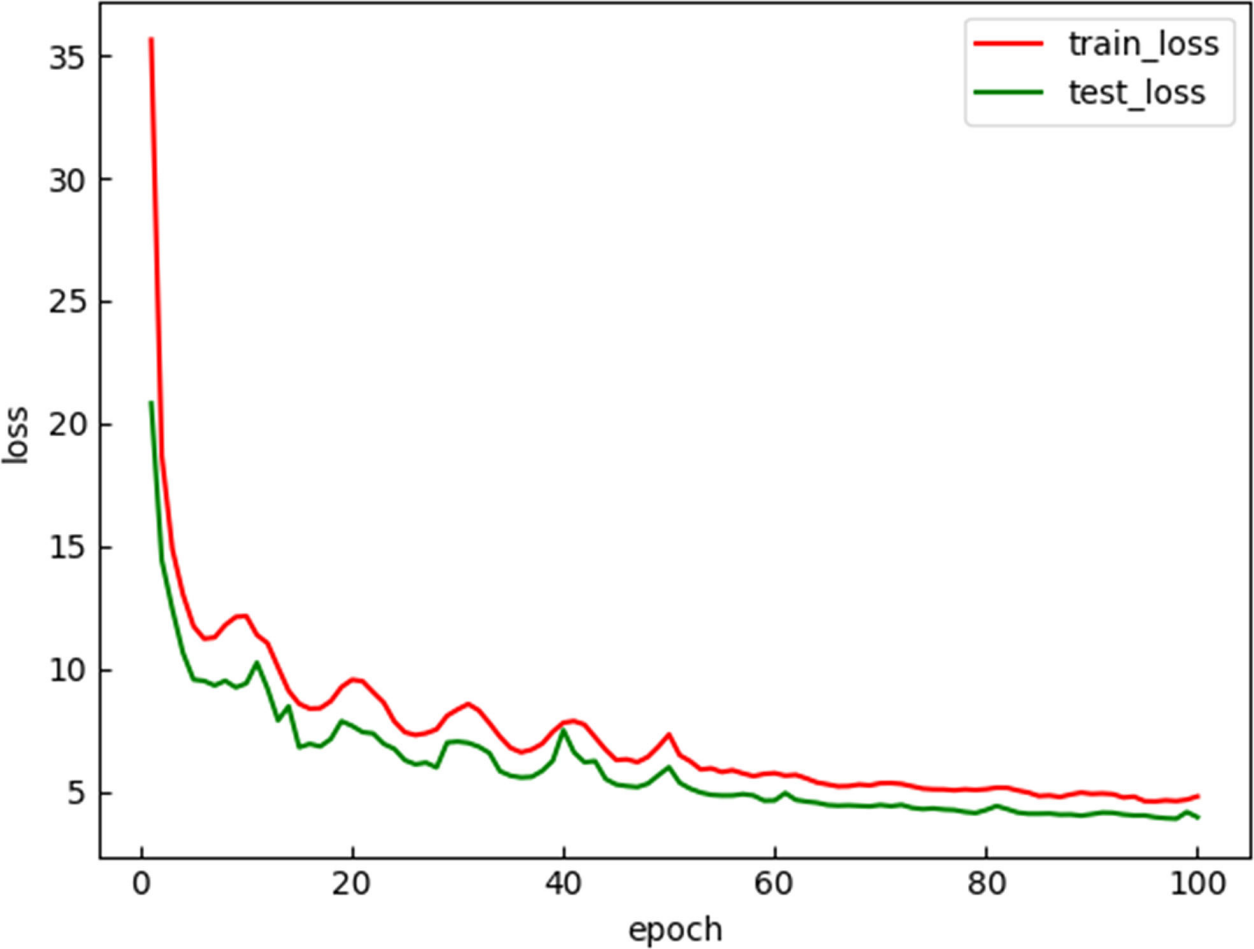
Loss value change curve of the training set and the test set.

This study uses Pytorch to improve the YOLOv4. Graphics processor unit (GPU) is NVIDIA Quadro P2200 16 G, and the central processing unit (CPU) is Intel(R) Xeon(R) Silver 4210R. The training and improvement of YOLOv4 model are carried out on Windows 10 operating system. The momentum of the momentum optimizer in the network is set to 0.9, the initial learning rate of the weight is set to 0.001, the attenuation coefficient is set to 0.0005, and the number of training iterations is 100. The loss curves for training and test sets are shown in **Figure 7**.

#### 3.3.2 Pitaya fruit recognition network training

**Figure 8:**
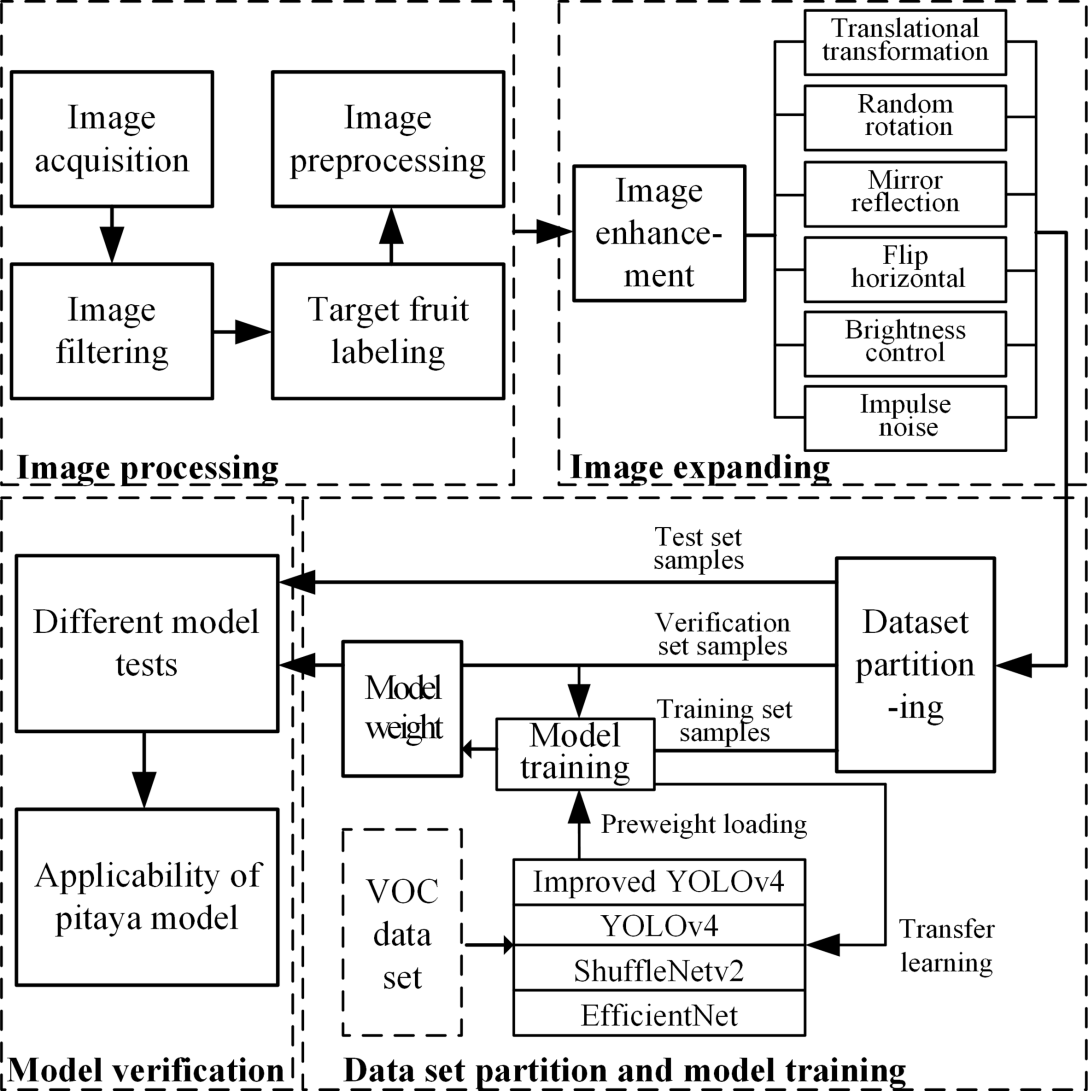
Flow chart of pitaya targets detection network.

The flow chart of target detection network is shown in **Figure 8**. The model effect is verified on the same verification set by comparing different improved models.

#### 3.3.3 Model evaluation indicators

In this paper, Precision (P), Recall (R), F1, AP, detection speed and weight size are selected as model evaluation indexes. Since only the pitaya fruit in the image needs to be identified, the pitaya fruit is regarded as a positive sample. On the contrary, all other objects are regarded as negative sample.


(12) $$P=\frac{TP}{FP+TP}$$

(13) $$R=\frac{TP}{FN+TP}$$

(14) $$F1=\frac{2RP}{R+P}$$

(15) $$AP=\int_{0}^{1}P(R)dR$$

Among them, the meaning of TP, FP and FN are as follows:

TP: The number of positive samples correctly identified, which is the correct rate.
FP: The number of positive samples incorrectly identified, which is the error rate.
FN: The number of positive samples missed, which is the omission rate.

## 4 Results and analysis

### 4.1 Comparison of detection results of different backbone networks

In order to prove the superiority of the improved model under GhostNet framework, this paper conducted comparative experiments on the traditional YOLOv4 and different backbone networks. CSPDarkNet53, GhostNet, ShuffleNetV2, and EfficientNet were used as backbone networks to detect Precision, Recall, F1, AP, detection speed and weight size of pitaya fruit targets. The comparison results of the detection performance of different backbone networks are shown in **Table 1**. As can be seen from **Table 1**, although Precision, Recall, F1, and AP of the models with GhostNet, ShuffleNetV2, and EfficientNet as the backbone networks have been decreased, the detection speed has been improved, and the weight size has been reduced significantly. Among them, the weight size of the model with GhostNet and ShuffleNetV2 as the backbone network is similar, but in terms of detection speed, the model with GhostNet as the backbone network is the fastest and the comprehensive effect is the best.

**Table 1:** Comparison results of detection performance of different backbone networks.

Backbonenetworks	IoU score	Precision/%	Recall/%	F1 score/%	Average precision/%	Detection speed/frames·s^-1^	Weight size/MB
CSPDarkNet53	0.50	92.43	92.22	92	94.34	27.4	244
	0.75	62.16	59.67	61	54.76		
GhostNet	0.50	91.32	87.30	88	92.10	32.6	152
	0.75	60.47	59.03	58	52.05		
ShuffleNetV2	0.50	91.38	87.50	89	92.16	30.2	151
	0.75	60.85	59.07	59	52.11		
EfficientNet	0.50	90.86	86.91	86	91.72	29.8	163
	0.75	60.03	58.40	57	51.78		

### 4.2 Analysis of pitaya fruit identification results

**Figure 9:**
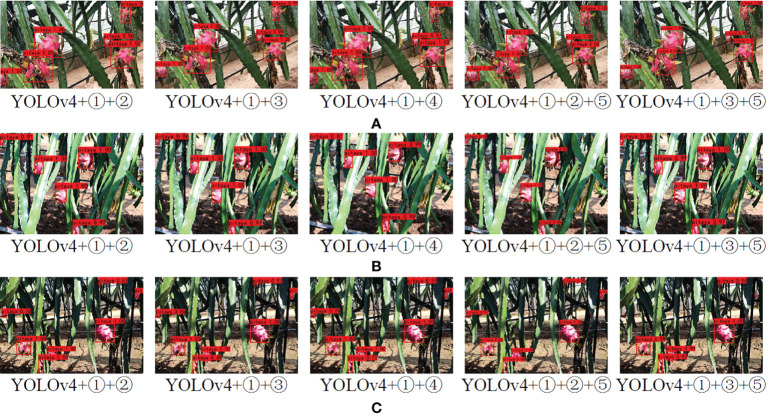
Different recognition algorithms for pitayas in three scenes: **(A)** Rainy weather; **(B)** Occlusion; **(C)** Backlighting.

The network structure of this paper was based on YOLOv4, it used GhostNet as the feature extraction backbone network, applied the improved combinational convolution-CA module to the feature fusion, and referenced the Ghost Module in Yolo Head. In order to prove the superiority of the improved network based on YOLOv4, it was necessary to compare and analyze the performance of the detection network before and after the improvement. The pitaya fruit recognition experiment was performed on YOLOv4 and the improved YOLOv4 network under the pitaya fruit dataset. The specific improvements were as follows: ①Replacement of the backbone network. GhostNet was used as the backbone network. ②The improved combinational convolution-CA module was used at feature fusion ac. ③The improved combinational convolution-CA module was used at feature fusion bd. ④The improved combinational convolution-CA module was used at feature fusion abcd. ⑤Ghost Module was referenced in Yolo Head. Effects of five improved methods on recognition of pitaya fruit in different natural environment as shown in **Figure 9**. Comprehensive comparison showed that GhostNet was used as the backbone network, the improved combinational convolution-CA module was used at feature fusion ac, and the improved algorithm of Ghost Module was referenced in Yolo Head to detect pitaya fruit in rainy days, occlusion and backlight conditions. It had high recognition ac-curacy, while the other four target detection networks had missed detection and false detection, and the recognition accuracy was lower than that of the YOLOv4+①+②+⑤ net-work structure model. Therefore, the improved algorithm in this paper has strong robustness and can adapt to different situations in natural environment.

The comparison results of the detection performance of five different improved algorithms are shown in **Table 2**. It can be seen from **Table 2** that Precision, Recall, F1, AP and detection speed of the YOLOv4+①+②+⑤ network structure model are higher than those of the other four target detection networks. When IoU is 0.50, these indexes were 99.23%, 95.10%, 98%, 98.94%, and 37.2 frames·s^-1^, and the weight size was the smallest, which was 59.4MB, which proved that the model was significantly better than the other four network structures. Compared with the results of traditional YOLOv4, when IoU is 0.50, Precision was increased by 6.8 percentage points, Recall was increased by 2.88 percentage points, F1 was increased by 6 percentage points, AP was increased by 4.6 percentage points, the detection speed was increased by 9.8 frames·s^-1^, and the weight size was reduced by 184.6MB.

**Table 2:** Comparison results of detection performance of different improved networks.

Algorithms	IoU score	Precision/%	Recall/%	F1 score/%	Average precision/%	Detection speed/frames·s^-1^	Weight size/MB
YOLOv4+①+②	0.50	97.15	92.86	95	97.38	36.4	60.7
	0.75	79.47	76.18	77	74.50		
YOLOv4+①+③	0.50	96.40	89.72	93	96.88	35.9	81.1
	0.75	80.01	75.51	77	72.64		
YOLOv4+①+④	0.50	96.35	89.75	93	96.86	35.7	99.4
	0.75	78.50	74.81	76	72.58		
YOLOv4+①+②+⑤	0.50	99.23	95.10	98	98.94	37.2	59.4
	0.75	80.00	76.19	78	74.62		
YOLOv4+①+③+⑤	0.50	97.17	92.48	95	97.39	36.8	79.9
	0.75	79.51	76.53	78	74.56		

Also, parameters, Flops, and MAC are frequently used when evaluating the size and computational complexity of deep learning models. Parameters represent the total number of parameters inside the model, which is used to measure the size of the model. Flops is the number of floating-point operations, which is used to measure the computational complexity of the model. MAC is the memory access cost, which is used to evaluate the memory usage of the model at runtime. The comparison results of traditional YOLOv4 and the above five different improved algorithms are shown in **Table 3**. It can be seen from **Table 3** that the total parameters of the YOLOv4+①+②+⑤ network structure model are the smallest, the model computational complexity is the lowest, and the model running memory is the least.

**Table 3:** Comparison results of different improved networks.

Algorithms	Parameters	Flops/G	MAC/MB
YOLOv4	64040001	29.95	606.54
YOLOv4+①+②	15839686	13.88	578.49
YOLOv4+①+③	21204446	13.88	582.72
YOLOv4+①+④	25976382	20.7	600.26
YOLOv4+①+②+⑤	15503686	13.61	578.49
YOLOv4+①+③+⑤	20868446	13.61	582.72

**Figure 10:**
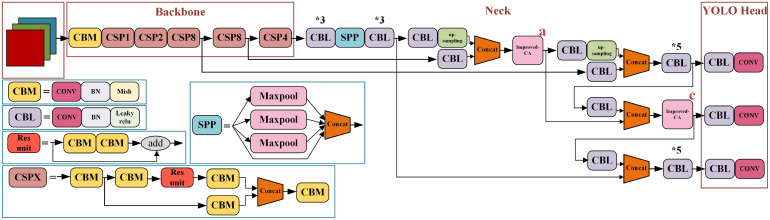
The improved YOLOv4 network structure diagram. * means repeat the operation.

In conclusion, the improved YOLOv4 structure proposed in this paper is shown in **Figure 10**, which can effectively identify pitaya fruit in natural environment and meet the requirements of target recognition accuracy and speed, with the best overall performance.

## 5 Conclusions and discussion

This paper proposed an improved YOLOv4 target recognition algorithm. It used GhostNet as the backbone network, used the improved combinational convolution-CA module at feature fusion, and referenced the Ghost Module in Yolo Head. The algorithm was lighter than the traditional YOLOv4, with faster detection speed and higher recognition accuracy. The model had high recognition accuracy when detecting pitaya fruit in rainy days, occlusion and backlight conditions.

Compared with the results of traditional YOLOv4, when IoU is 0.50, the accuracy of the improved YOLOv4 target recognition algorithm proposed in this paper on the augmented dataset can reach 99.23%, and the weight size is about 1/4 of the traditional YOLOv4. The average accuracy is improved by nearly 5 percentage points, and the detection speed is improved by nearly 10 frames·s^-1^. Experiments prove that the YOLOv4 recognition algorithm proposed in this study that was combined CA with the improved combinational convolution has significant advantages.

The method is suitable for recognition of other fruits and even other kinds of objects. In future work, it is applied to the robot platform, and the corresponding robotic arm, manipulator, and binocular camera are equipped on its chassis to complete the entire picking process.

## Data availability statement

The raw data supporting the conclusions of this article will be made available by the authors, without undue reservation.

## Author contributions

Conceptualization, FZ and WC. Methodology, WC and SW. Software, WC and SW. Validation, FZ, WC and SW. Formal analysis, FZ. Investigation, WC, XC and XW. Resources, WC and SW. Data curation, FZ and WC. Writing—original draft preparation, FZ and WC. Writing—review and editing, FZ and WC. Visualization, FZ and WC. Supervision, FZ, NY and SF. Project administration, FZ and XZ. Funding acquisition, FZ and XZ. All contributed to the article and approved the submitted version.

## Funding

This research was funded by the Scientific and Technological Project of Henan Province (No. 212102110029), and the National Natural Science Foundation of China (No. 61771224), High-tech Key Laboratory of Agricultural Equipment and Intelligence of Jiangsu Province (No. JNZ201901) and the Colleges and Universities of Henan Province Youth Backbone Teacher Training Program (No. 2017GGJS062).

## Conflict of interest

The authors declare that the research was conducted in the absence of any commercial or financial relationships that could be construed as a potential conflict of interest.

## Publisher’s note

All claims expressed in this article are solely those of the authors and do not necessarily represent those of their affiliated organizations, or those of the publisher, the editors and the reviewers. Any product that may be evaluated in this article, or claim that may be made by its manufacturer, is not guaranteed or endorsed by the publisher.
