# Corrigendum: Improved YOLOv4 recognition algorithm for pitaya based on coordinate attention and combinational convolution

**DOI:** 10.3389/fpls.2022.1092374

**Published:** 2022-12-21

**Authors:** Fu Zhang, Weihua Cao, Shunqing Wang, Xiahua Cui, Ning Yang, Xinyue Wang, Xiaodong Zhang, Sanling Fu

**Affiliations:** ^1^ College of Agricultural Equipment Engineering, Henan University of Science and Technology, Luoyang, China; ^2^ Collaborative Innovation Center of Machinery Equipment Advanced Manufacturing of Henan Province, Henan University of Science and Technology, Luoyang, China; ^3^ School of Electrical and Information Engineering, Jiangsu University, Zhenjiang, China; ^4^ Key Laboratory of Modern Agricultural Equipment and Technology of Ministry of Education, Jiangsu University, Zhenjiang, China; ^5^ College of Physical Engineering, Henan University of Science and Technology, Luoyang, China

**Keywords:** improved YOLOv4, GhostNet, coordinate attention, improved combinational convolution module, target recognition

In the published article, there was an error in **Figure 2** as published. An error appears in the upper left corner of the figure. The corrected **Figure 2** and its caption YOLOv4 network structure diagram. * means repeat the operation. appear below.

**Figure 2:**
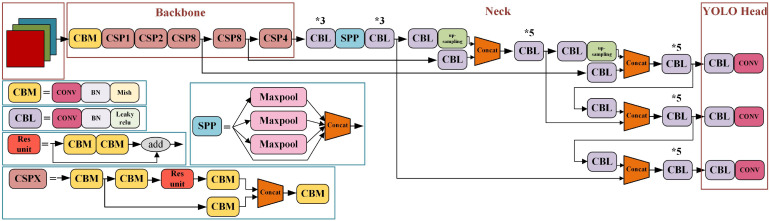
YOLOv4 network structure diagram. * means repeat the operation.

In the published article, there was an error in **Figure 6** as published. The left side of the figure is missing. The corrected **Figure 6** and its caption Improved combinational convolution-CA module at fusion. appear below.

**Figure 6:**
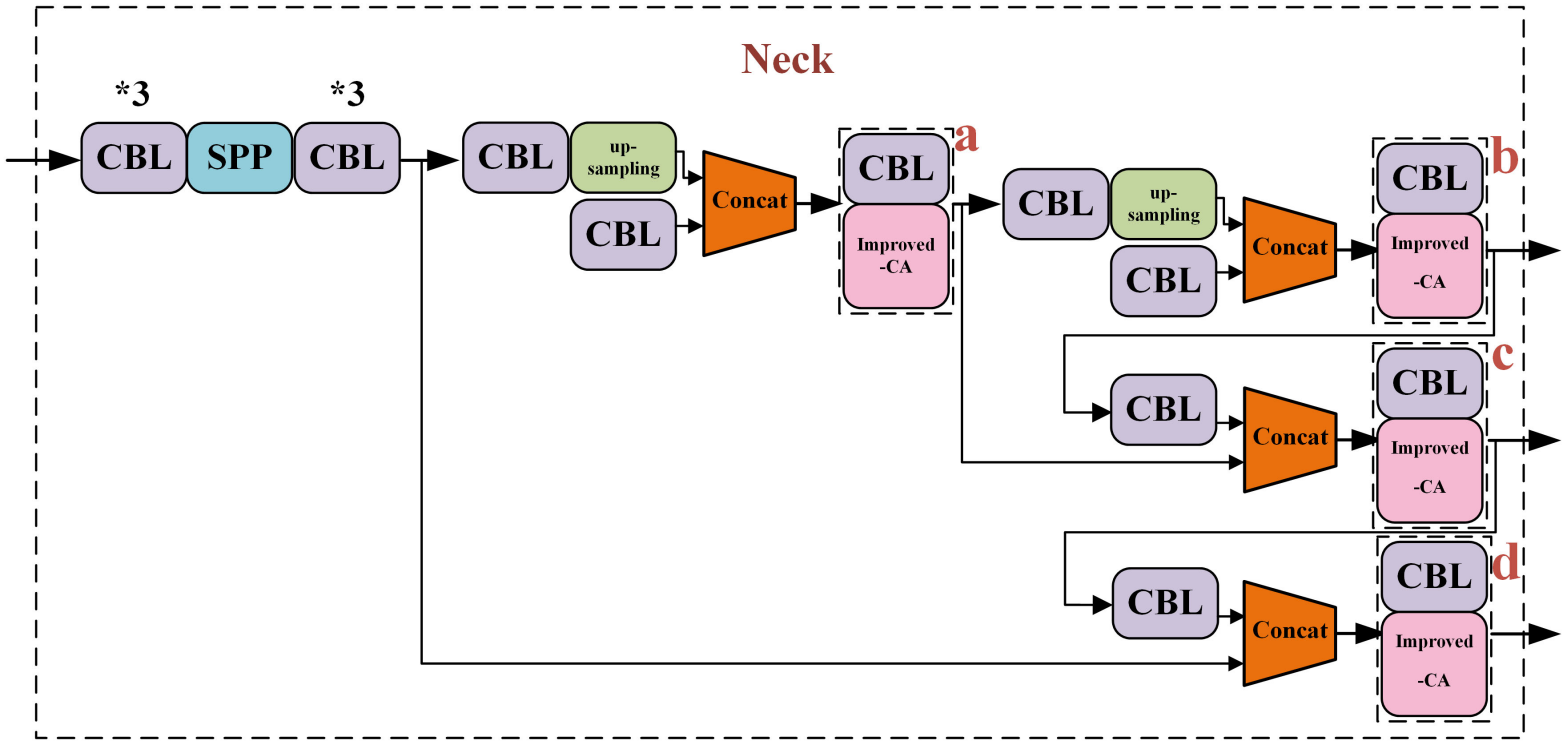
Improved combinational convolution-CA module at fusion.

In the published article, there was an error in **Figure 10** as published. An error appears in the upper left corner of the figure. The corrected **Figure 10** and its caption The improved YOLOv4 network structure diagram. * means repeat the operation. appear below.

**Figure 10:**
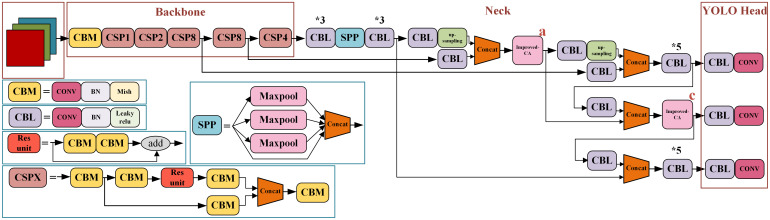
The improved YOLOv4 network structure diagram. * means repeat the operation.

The authors apologize for this error and state that this does not change the scientific conclusions of the article in any way. The original article has been updated.

